# Surfactin Shows Relatively Low Antimicrobial Activity against *Bacillus subtilis* and Other Bacterial Model Organisms in the Absence of Synergistic Metabolites

**DOI:** 10.3390/microorganisms10040779

**Published:** 2022-04-05

**Authors:** Lars Lilge, Nadine Ersig, Philipp Hubel, Moritz Aschern, Evelina Pillai, Peter Klausmann, Jens Pfannstiel, Marius Henkel, Kambiz Morabbi Heravi, Rudolf Hausmann

**Affiliations:** 1 Department of Bioprocess Engineering (150k), Institute of Food Science and Biotechnology (150), University of Hohenheim, Fruwirthstr. 12, 70599 Stuttgart, Germany; nadine.ersig@uni-hohenheim.de (N.E.); mo.aschern@gmx.de (M.A.); peterklausmann@gmail.com (P.K.); marius.henkel@uni-hohenheim.de (M.H.); kambiz_morabbi@yahoo.com (K.M.H.); rudolf.hausmann@uni-hohenheim.de (R.H.); 2 Core Facility Hohenheim, Mass Spectrometry Core Facility, University of Hohenheim, Ottilie-Zeller-Weg 2, 70599 Stuttgart, Germany; philipp.hubel@uni-hohenheim.de (P.H.); jens.pfannstiel@uni-hohenheim.de (J.P.); 3 Core Facility Hohenheim (640), Data Management & Bioinformatics, University of Hohenheim, Emil-Wolff-Str. 12, 70599 Stuttgart, Germany; pillai@uni-hohenheim.de

**Keywords:** *Bacillus subtilis*, surfactin, secondary metabolites, biosurfactants, stress response

## Abstract

Surfactin is described as a powerful biosurfactant and is natively produced by *Bacillus* *subtilis* in notable quantities. Among other industrially relevant characteristics, antimicrobial properties have been attributed to surfactin-producing *Bacillus* isolates. To investigate this property, stress approaches were carried out with biotechnologically established strains of *Corynebacterium glutamicum*, *Bacillus* *subtilis*, *Escherichia coli* and *Pseudomonas putida* with the highest possible amounts of surfactin. Contrary to the popular opinion, the highest growth-reducing effects were detectable in *B. subtilis* and *E.* *coli* after surfactin treatment of 100 g/L with 35 and 33%, respectively, while *P. putida* showed no growth-specific response. In contrast, other antimicrobial biosurfactants, like rhamnolipids and sophorolipids, showed significantly stronger effects on bacterial growth. Since the addition of high amounts of surfactin in defined mineral salt medium reduced the cell growth of *B.* *subtilis* by about 40%, the initial stress response at the protein level was analyzed by mass spectrometry, showing induction of stress proteins under control of alternative sigma factors σ^B^ and σ^W^ as well as the activation of LiaRS two-component system. Overall, although surfactin is associated with antimicrobial properties, relatively low growth-reducing effects could be demonstrated after the surfactin addition, challenging the general claim of the antimicrobial properties of surfactin.

## 1. Introduction

The soil bacterium *Bacillus subtilis* reveals a large number of proteins that enable the efflux of various biologically active components [1]. These include the class of lipopeptides as one of the most important groups of secondary metabolites produced by several *Bacillus* strains [2]. In the case of the established laboratory strain *B. subtilis* 168, lipopeptide production is facilitated by two operons (*srfAA-AD* for surfactin and *ppsA-E* for plipastatin) encoding nonribosomal peptide synthetases (NRPSs) [3,4]. Surfactin is the main lipopeptide in *B. subtilis* and is produced in several grams per litre under optimised conditions [5]. In adapted high cell-density fermentation bioprocesses using *B. subtilis* 3NA, an amount of about 26 g/L surfactin was produced [6]. To ensure controlled surfactin formation, several regulatory mechanisms are involved in lipopeptide production, such as ComX-mediated quorum sensing and nutrition state sensing regulators [7,8,9]. Posttranslationally, the peptidyl carrier protein domains of three surfactin synthetase subunits (SrfAA, SrfAB, SrfAC) need to be activated by the 4-phosphopantetheinyl transferase Sfp [10]. In particular, *B. subtilis* strain 168 exhibits a non-functional *sfp* version due to a single base duplication, which prevents surfactin formation [11].

Surfactin is characterised by a cyclic lactone structure consisting of a peptide moiety comprising seven amino acids (L-Glu, L-Leu, D-Leu, L-Val, L-Asp, D-Leu, L-Leu) combined with a β–hydroxy fatty acid of varying chain length from C_13_ to C_15_, with C_14_ and C_15_ usually being the predominant versions in *B. subtilis* [12,13]. Based on these structural characteristics, surfactin is reported to have a critical micelle concentration of about 15 mg/L [14]. In addition, antibacterial, antiviral, antitumor and haemolytic properties are attributed to surfactin [15,16,17,18]. In more detail, surfactin facilitates membrane destabilization through incorporation into lipid bilayers, chelation of cations and pore formation [19,20,21]. Previous studies have described antimicrobial activities against various pathogenic bacteria, such as *Enterococcus faecalis*, *Staphylococcus areus* and *Pseudomonas aeruginosa* [22]. In addition, efficacy against the phytopathogenic fungus *Fusarium verticillioides* [23] and the phytopathogenic bacterium *Pseudomonas syringae* [24] has been demonstrated. However, most studies report the effect of surfactin when using the whole *Bacillus* cell culture or the cell-free supernatant after cultivation, which also contain other antimicrobial metabolites such as fengycin, iturin or bacteriocins. Furthermore, other lipopeptides, including iturin and fengycin, can also be purified when surfactin is extracted from the cell-free supernatant, which can lead to combination effects when analysing the impact on microbes [25]. More efficient is the purification of the biosurfactant as described by Loiseau et al. [26] when antimicrobial and antibiofilm activity against *Legionella pneumophila* was described using purified surfactin from the contaminant *B. subtilis* strain AM1.

As a native production strain, *B. subtilis* displays strategies for tolerance to self-produced surfactin. In this context, the motive force-dependent efflux pump SwrC (YerP) has been described as a surfactin self-resistance protein involved in surfactin efflux [27]. In the presence of foreign biosurfactants, *B. subtilis* shows different regulatory systems for a flexible stress response. For friulimicin B, a cyclic lipopeptide produced by *Actinoplanes friuliensis*, and daptomycin, a cyclic lipodepsipeptide, Wecke et al. [28] showed that daptomycin exclusively stimulates the LiaRS two-component system, although the compounds are structurally similar antibiotics and induce cell envelope stress [28].

In comparison, treatment with rhamnolipid, a rhamnose-containing glycolipid [29] produced by the Gram-negative pathogen *Pseudomonas aeruginosa*, activates the alternative sigma factor σ^M^ and the LiaRS and CssRS two-component systems in *B. subtilis* [30]. Rhamnolipids are composed of either a single or a pair of β-hydroxyl fatty acids with a varying chain length between C_8_ and C_16_ (C_10_ as the dominant version in *P. aeruginosa*) linked by the number of rhamnose units (mono- or di-rhamnolipid) [31]. This structure leads to a critical micelle concentration of about 25 mg/L [14]. As rhamnolipids are described as another type of bioactive molecule, several applications in the biomedicial therapeutic and agriculture sectors may be possible in the future [32].

Another well-known biosurfactant that represents an important class of antimicrobial glycolipids is sophorolipid, which is produced by yeasts such as *Starmerella bombicola* [33,34]. Several studies have demonstrated both antimicrobial properties on planktonic *B. subtilis* cells and inhibitory effects on corresponding biofilms [35,36,37]. Sophorolipids are composed of a sophorose part associated by β-1,2 glycosidic linkages with hydroxy fatty acids (C_16_–C_18_) as the hydrophobic part of this biosurfactant. Furthermore, the fatty acid chain can be modified by acetylation and lactonization [38]. Especially the lactonised version shows the ability to reduce surface tension and biological activity [39]. The biosurfactant activity was also clarified by describing a critical micelle concentration of about 70 mg/L [14].

In this report, the overall antimicrobial effect of surfactin on the biotechnologically established bacteria *Bacillus subtilis*, *Corynebacterium glutamicum*, *Escherichia coli* and *Pseudomonas putida* was compared with other antimicrobial biosurfactants, namely rhamnolipids and sophorolipids. In this way, the antimicrobial properties of surfactin for different Gram-positive and -negative bacterial species were compared and the occurring effects on bacterial growth were confronted with other bioactive biosurfactants that have been described with antimicrobial characteristics. Since derivatives of *B. subtilis* strain 168 with a functional *sfp* gene are native surfactin producers [6,40,41], the initial proteome adaptation of the non-producing *B. subtilis* strain KM0 (strain 168; *trp*+), as there is consequently no preadaptation to surfactin, was analysed after treatment with high amounts of surfactin using mass spectrometric approaches. The findings on the specific proteome response after initiation of surfactin stress will increase the knowledge on the adaptation of the production strain to high amounts of surfactin and lead to the identification of genetic engineering targets for improved production strains.

## 2. Materials and Methods

### 2.1. Bacterial Strains and Cultivation Conditions

Chemicals were all obtained from Carl Roth GmbH & Co. KG (Karlsruhe, Germany) as long as not otherwise stated. The strains used in this study are shown in Table 1. Pre-cultures and main cultures for comparative stress approaches were conducted in LB medium (5 g/L yeast extract, 5 g/L NaCl and 10 g/L tryptone). Therefore, pre-cultures were inoculated with a glycerol stock of the respective strain and cultivated overnight. The main culture (50 mL) was inoculated with exponentially growing cells (approximately 2.5% in relation to the main culture medium) with an initial optical density (OD_600_) of 0.1. In the case of the *B. subtilis* cultures used for surfactin supplementation cultivations as well as the stress approaches for proteomic analysis, the cultures were cultivated in mineral salt medium (MSM) [42]. The medium consisted of 8 g/L glucose, 4.0 × 10^−6^ M Na_2_EDTA × 2 H_2_O, 4.0 × 10^−6^ M FeSO_4_ × 7 H_2_O, 7.0 × 10^−6^ M CaCl_2_, 1.0 × 10^−6^ M MnSO_4_ × H_2_O, 0.05 M (NH_4_)_2_SO_4_, 0.04 M Na_2_HPO_4_ × 2 H_2_O, 0.03 M KH_2_PO_4_ and 8.0 × 10^−4^ M MgSO_4_ × 7 H_2_O. All cultivations were carried out in baffled shaking flasks at 30 °C for *P. putia* and *C. glutamicum* or 37 °C for *E. coli* and *B. subtilis* and 120 rpm as biological duplicates for stress approaches and biological triplicates for mass spectrometric analyses.

### 2.2. Stress Approach

After reaching an OD_600_ of approximately 1, the main culture was split and equal volumes of the cell suspension and the biosurfactant solution were transferred to a pre-warmed 100 mL shake flask. Surfactin solutions were prepared by dilution of surfactin powder (*B. subtilis* produced; >90% purity; information on the isoforms is provided in the Supplemental Data) obtained from KANEKA (Osaka, Japan). To evaluate the effect of surfactin treatment on protein expression, the solutions were prepared in mineral salt medium and the effect was evaluated by proteome analysis. To evaluate the antimicrobial activity, surfactin and rhamnolipid (*P. aeruginosa* produced; 90% purity; AGAE Technologies, Corvallis, OR, USA) solutions were prepared in distilled water, whereas sophorolipids (di-acetylated lactonic sophorolipid C18:1 ω-1; 96.9% purity; Amphi-Star, Ghent, Belgium) were dissolved in 50% (*v*/*v*) ethanol. All biosurfactant solutions were filter-sterilised before use in the stress approaches.

For mass spectrometry (MS), the cell suspension was transferred to MSM supplemented with 75 g/L surfactin. Samples (5 mL) were collected immediately before and 10 min after stress initiation. After centrifugation (13,700 rpm, 3 min), cell pellets were mixed in 1 mL cell lysis buffer (2% sodium dodecyl sulfate, 20 mM dithiothreitol (DTT) and 150 mM Tris-HCl (pH 8.5)) and incubated at 95 °C for 5 min. The lysates were stored at −80 °C until the proteome was analysed by MS.

### 2.3. Pretreatment for MS Analysis

Samples were centrifuged (15 min, 13,700 rpm, 4 °C), the supernatant was separated and the proteins were precipitated with chloroform and methanol [46]. Subsequently, protein pellets were solubilised in 6 M urea and 50 mM Tris-HCl (pH 8.5) followed by determination of protein concentrations using the Bradford assay [47]. Afterwards, cysteine reduction was performed by addition of a final concentration of 10 mM DTT to 25 µg of protein solution. Samples were incubated for 30 min at 56 °C and 1000 rpm. Cysteine alkylation was done via addition of 30 mM iodoacetamide and incubation for 20 min at room temperature (RT) under dark conditions. Alkylation was stopped when 50 mM DTT was added and another incubation for 10 min at RT. Proteins were digested overnight at 30 °C using 0.5 µg LysC protease (Roche, Basel, Switzerland) in 50 mM Tris-HCl (pH 8.5). Next, urea was then diluted to 2 M in the reaction mixture with the addition of a respective volume of 50 mM Tris-HCl (pH 8.5) and 1 µg trypsin (Roche) for further protein digestion (4 h at 37 °C). Digestion was inhibited by adding 3 µL 10% TFA (trifluoroacetic acid). Finally, peptide mixtures were desalinated and concentrated using C18 stage tips [48]. After the samples dried under vacuum and were dissolved in 20 µL of 0.1% TFA, nanoLC-MS/MS analyses were performed with aliquoted quantities of 1.5 µL.

### 2.4. NanoLC-MS/MS Analysis

NanoLC-ESI-MS/MS analyses were conducted on an Ultimate 3000 nano-RSLC (Thermo Fisher Scientific, Waltham, MA, USA) connected to a Q-Exactive HF-X mass spectrometer (Thermo Fisher Scientific) equipped with a Nanospray-Flex ion source (Thermo Fisher Scientific). For desalination and concentration of peptides, trap column (5 mm × 30 µm, Thermo Fisher Scientific) was used and separation was done with a 25 cm × 75 µm nanoEase MZ HSS T3 reversed phase column (100 Å pore size, 1.8 µm particle size, Waters, Milford, MA, USA) at a constant temperature of 35 °C. Separation of peptides was performed at a flow rate of 300 nL/min applying a 90 min gradient with the following profile: 2–15% solvent B in 37 min, 15–30% solvent B in 30 min, 30–45% solvent B in 13 min and 45–55% solvent B in 10 min. Solvents used were 0.1% formic acid (solvent A) and 0.1% formic acid in acetonitrile/H_2_O (80/20, *v*/*v*, solvent B).

The Q Exactive HF-X was operated using the XCalibur 4.1.31.9 software. MS spectra (*m*/*z* = 300–1800) were obtained in the Orbitrap with a resolution of 60,000 (*m*/*z* = 200) with a 100 ms maximum injection time (MIT) and an automatic gain control (AGC) value of 1 × 10^6^. Calibration of the Orbitrap analyser internally was performed using lock-mass ions from ambient air as described in Olsen et al. [49]. MS/MS spectra of the top 30 peptide precursors per cycle were generated in the Orbitrap using high energy collision dissociation (HCD) fragmentation with a resolution of 15,000 and a normalised collision energy of 27. Other MS/MS spectra settings were an 1.6 Da isolation width, an MIT of 100 ms and an automatic gain control (AGC) value of 5 × 10^5^.

### 2.5. MS Data Analysis and Protein Quantification

Raw files were implemented in MaxQuant [50] version 1.6.2.10 for identification and label-free quantification (LFQ) of proteins. Therefore, MaxQuant was carried out with the Andromeda database search engine [51]. MS spectra and MS/MS spectra were compared with the protein sequence database of *Bacillus subtilis* (strain 168) from UniProt [52]. Frequently occurring contamination sequences and reversed sequences as decoy databases were added automatically by MaxQuant. Mass tolerances of 4.5 ppm (parts per million) were used for MS spectra and 20 ppm for MS/MS spectra. Trypsin was indicated as enzyme present and allowed two missed cleavages. Carbamidomethylation of cysteines was defined as a fixed modification, and N-terminal acetylation of proteins as well as methionine oxidation were accepted as variable modifications. The ‘match between runs’ function of MaxQuant was used with a match time window of one minute and for an alignment, a time window of 20 min was applied. The thresholds of peptide false discovery rate (FDR) and protein FDR thresholds were defined to be 0.01.

Two samples Welch’s *t*-test and Volcano plots were generated by Perseus version 1.6.14.0 [53]. Matches containing contaminations (e.g., keratins, trypsin), with reverse databases and candidates only identified by site in MaxQuant were rejected from further analysis. First, normalised LFQ values from MaxQuant were log2 transformed. Missing values were imputed with random numbers from a normal distribution using a width of 0.3 and a downshift of 1.8. Significant changes in protein abundance were analysed using a Welch’s *t*-test for two samples with a permutation-based FDR at a cut-off of 0.05 and an S0 value of 1. Volcano graphs were applied for the comparison of sample groups.

LFQ intensities of the MS data were fed into the R package *proteus* (v. 0.2.14) [54] for differential expression analysis. Briefly, reverse hits, identifications only by site and potential contaminants were removed in advance, LFQ intensities were log2 transformed, filtered by at least three occurrences in at least one condition and analysed by the wrapper *limmaDE*, which is included in the *proteus* package. The statistical significance level for the rejection of the null hypothesis was defined as 0.05. Protein annotation to their functional category has been acquired from SubtiWiki database [55]. This annotation has been used for the generation of the Voronoi treemaps by mapping over gene names.

The proteomics data provided by the mass spectrometric analyses were submitted to the ProteomeXchange Consortium via the PRIDE [56] partner repository with the dataset identifier PXD029668.

### 2.6. Voronoi Treemap Generation

The plotting and treemap creation have been performed using the Java-Portlet *Voronoi-Treemap-Portlet* provided by the Quantitative Biology Center in Tübingen [57]. It uses a java library, which computes voronoi treemaps based on the algorithm by Nocaj and Brandes [58]. Applied on the provided SubtiWiki hierarchy, the weight of each polygon is set by the occurrences of the measured protein in the whole dataset. The color shading reflects the change of protein expression by log2 fold-changes.

## 3. Results

### 3.1. Effect of Surfactin Treatment on Bacterial Cell Growth

While it is already known that Gram-positive bacteria show a higher sensitivity to biopharmaceuticals than Gram-negative bacteria, it is imperative to determine an overall effect of surfactin as an antimicrobial biosurfactant against a variety of microbes. Accordingly, model organisms or well-established representatives used in both fundamental research and biotechnological applications were employed for initial stress approaches with high surfactin amounts (Table 1). For more insights into the surfactin used in the stress approaches, mass spectrometric analyses were carried out. More specifically, the most abundant surfactin version exhibited the well-known peptide structure E-L-L-V-D-L-L. In this context, the highly active surfactin C isoform comprised about 28%. A more detailed overview of the quantitative mass spectrometric analyses of the surfactin isoforms is provided in the Supplementary Data. To test the effect of surfactin solutions on both types of bacteria, the Gram-positive *Bacillus subtilis* and *Corynebacterium glutamicum* and the Gram-negative *Escherichia coli* and *Pseudomonas putida* were used.

**Figure 1:**
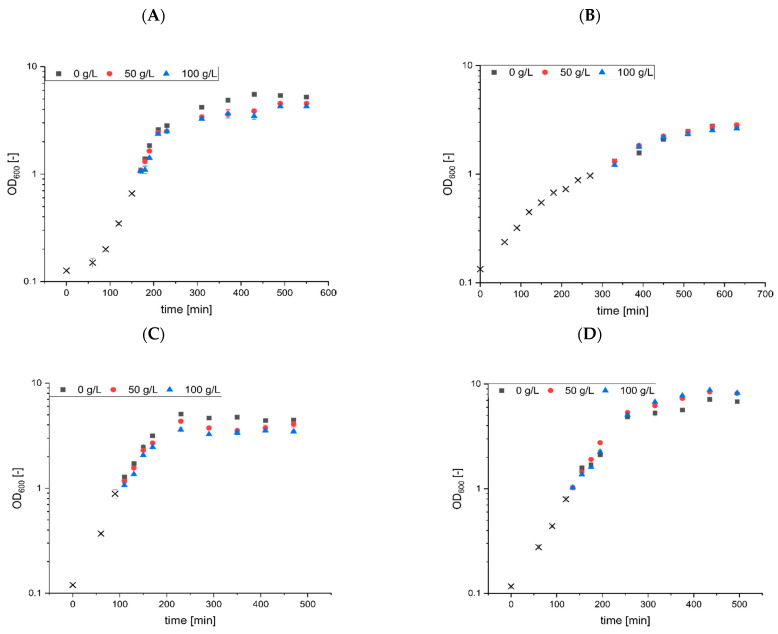
Surfactin stress approaches with biotechnologically established strains. Cell growth of *B. subtilis* KM0 (**A**), *C. glutamicum* ATCC13032 (**B**), *E. coli* BL21 (DE3) (**C**) and *P. putida* KT2440 (**D**) was monitored in LB medium. When the cultures reached an OD600 of approximately 1 (black crosses), equal volumes of cell culture were mixed with increasing concentrations of surfactin: 0 g/L (black squares), 50 g/L (red dots) and 100 g/L (blue triangles). Cell growth was monitored for further 6 h after stress induction. All stress approaches were performed in biological triplicates.

To ensure relatively comparable bacterial growth without limitations, the cultivations were carried out in LB medium. After reaching an optical density of about 1, surfactin solution dissolved in distilled water was added to the respective cell suspensions (Figure 1, please see Supplementary Material). After surfactin treatment, a relatively small but detectable reduction in growth rate was measured for *B. subtilis* and *E. coli*, while no effect was observed for *C. glutamicum* and a slightly stimulatory effect for *P. putida*. In detail, untreated *B. subtilis* cultures reached an OD_600_ of 5.2, while the addition of 50 and 100 g/L surfactin reduced the final OD_600_ to 4.5 and 4.3, respectively, corresponding to a biomass reduction of 13 to 17%. For the other Gram-positive bacterium, *C. glutamicum*, no effects on cell growth were observed, resulting in OD_600_ values of 2.83 (50 g/L surfactin) and 2.63 (100 g/L surfactin) compared to an unaffected OD_600_ of 2.77. Comparable to *B. subtilis*, *E. coli* cultures showed a reduction in OD_600_ values of about 9 and 22% after addition of 50 and 100 g/L surfactin, respectively. In contrast, absolutely no negative effect was observed for *P. putida* cell growth, resulting in slightly increased optical densities of about 20% for 50 and 100 g/L surfactin.

In summary, only for *B. subtilis* and *E. coli* a slight reduction in cell growth was observed after addition of high amounts of surfactin, while absolutely no effect (*C. glutamicum*) or even a stimulating effect (*P. putida*) was observed. Since relatively high surfactin concentrations were used for the stress approaches, surfactin appears to have only low antimicrobial properties.

### 3.2. Comparative Analyses of Microbial Sensitivity to Rhamnolipids and Sophorolipids

**Figure 2:**
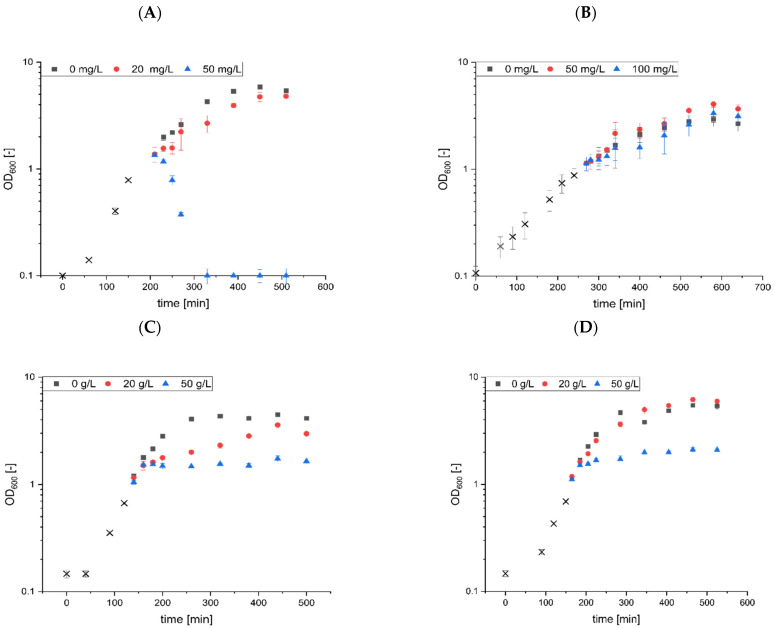

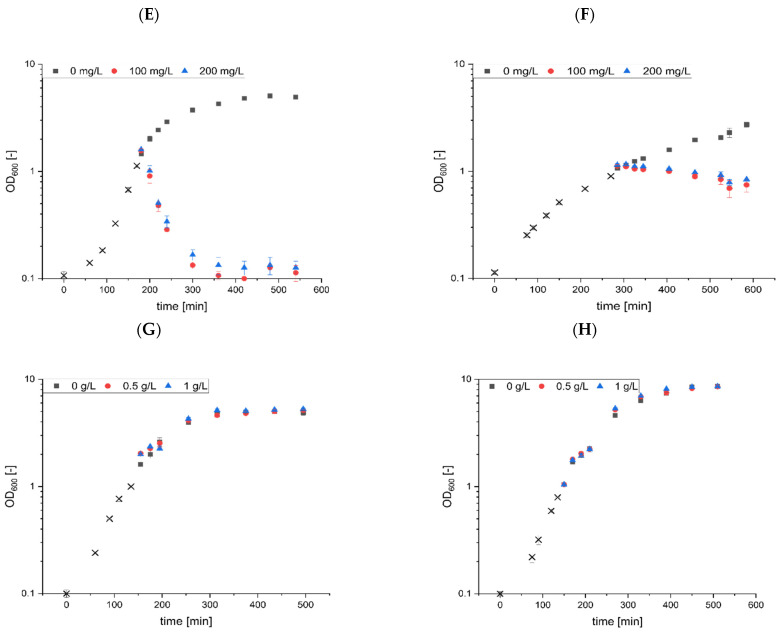
Stress approaches with biotechnologically established strains using rhamnolipids and sophorolipids. Cell growth of *B. subtilis* KM0 (**A**,**E**), *C. glutamicum* ATCC13032 (**B**,**F**), *E. coli* BL21 (DE3) (**C**,**G**) and *P. putida* KT2440 (**D**,**H**) was monitored in LB medium. After reaching an OD_600_ of approximately 1 (black crosses), increasing concentrations of rhamnolipids (**A**–**D**) or sophorolipids (**E**–**H**) were added to equal volumes of the cell culture. Cell growth was monitored for further 6 h after stress induction. All stress approaches were performed in biological triplicates.

While the surfactin stress approaches showed that the addition of high amounts of surfactin led to a slight reduction in cell growth for some strains, the surfactin concentrations used were not comparable to minimal inhibitory concentrations of other bioactive metabolites described for their antimicrobial properties. For validation, rhamnolipids and sophorolipids were used in comparable stress approaches as previously described for surfactin (Figure 2, please see Supplementary Material).

Compared to surfactin, a broad spectrum of microbial tolerances and sensitivities was determined through the stress approaches with rhamnolipids and sophorolipids as further exemplary bioactive metabolites. Specifically, rhamnolipids at a final concentration of 50 mg/L show a bactericidal effect for *B. subtilis*, while a concentration of 20 mg/L also showed an effect on *B. subtilis* cell growth (Figure 2A). No cell lysis was observed for the other Gram-positive representative, *C. glutamicum*. While rhamnolipid concentrations of 50 mg/L showed absolutely no effect, 100 mg/L rhamnolipids reduced cell growth for the next 4 h of cultivation before a comparable optical density was reached (Figure 2B).

With respect to *E. coli* and *P. putida*, heterologous production of rhamnolipids by genetically modified strains has been demonstrated in several studies [59,60]. However, in *E. coli*, both rhamnolipid treatments at 20 and 50 g/L resulted in a bacteriostatic effect. More specifically, significantly reduced cell growth was observed after addition of 20 g/L, resulting in comparable maximum optical densities relative to untreated cultures after a delay of 3 h. In contrast, no further cell growth was detected in *E. coli* treated with 50 g/L rhamnolipids. In *P. putida*, no change in cell growth was observed after treatment with 20 g/L rhamnolipids, while a bacteriostatic effect and a reduction in cell growth of about 61% was detected with 50 g/L.

In studies with sophorolipids, a clear difference in tolerance was observed between Gram-positive and Gram-negative bacteria. In particular, no effects were observed for *E. coli* and *P. putida* at sophorolipid concentrations of up to 1 g/L. In comparison, cell growth of *B. subtilis* was affected at both 100 mg/L and 200 mg/L of sophorolipids, resulting in cell lysis within the next 1 h. In *C. glutamicum*, treatment of 100 mg/L and 200 mg/L sophorolipids induced an arrest of cell growth, followed by a slight decrease in optical density over time. Overall, although both Gram-positive microorganisms displayed impaired cell growth, *C. glutamicum* appears to use different defence strategies against antimicrobial biosurfactants compared to *B. subtilis*.

Regarding *B. subtilis*, which seems to be the most sensitive bacterial strain in this comparative study, it appeared particularly clear that a multiple less of the substance amount of rhamnolipid (50 mg/L) and sophorolipid (100 mg/L) was required not only to reduce cell growth but also to induce cell lysis, as demonstrated by a noticeable reduction in optical density. In comparison, only a reduction of *B. subtilis* cell growth of about 17% was measured after addition of 100 g/L surfactin. To simulate this growth-reducing effect of accumulating surfactin during *B. subtilis* bioprocesses, a mineral salt medium used in bioreactor fermentrations for surfactin production [6] was applied to reproduce the effect on *B. subtilis* growth in combination with pre-existing surfactin concentrations.

### 3.3. Effect of Surfactin Present during the Cultivation Process of B. subtilis

**Figure 3:**
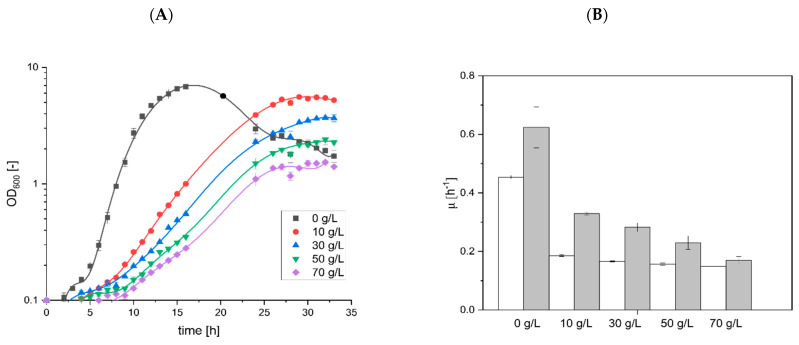
Effect of surfactin on *B. subtilis* during cultivation in defined mineral salt medium. (**A**) The *B. subtilis* KM0 strain was cultivated in mineral salt medium containing 8 g/L glucose. In addition, different surfactin concentrations of 0 (black squares), 10 (red dots), 30 (blue triangles), 50 (green inverted triangles) and 70 g/L (violet diamonds) were added to the cultivation medium. Cell growth was monitored hourly. A polynomial curve fit of the order of 9 was integrated using the Origin graphing tool. (**B**) The effect of the surfactin present during cultivation was determined using the overall growth rates calculated for the exponential growth phase (white bars) and the maximum specific growth rates during the cultivation process (grey bars). All cultivations were performed in biological triplicates.

In several studies, the production of surfactin was carried out in defined mineral salt medium [6,40,42]. To simulate the effect of surfactin accumulated during *B. subtilis* cultivation, increasing amounts of surfactin were added to main cultures with *B. subtilis* KM0 using 8 g/L of glucose (Figure 3, please see Supplementary Material). In the reference process, a maximum OD_600_ of 6.8 and a growth rate µ of 0.454 were achieved during the exponential phase of cultivation before glucose was depleted and the OD_600_ decreased [61]. After an extended lag phase, a similar maximum OD_600_ value was observed during cultivation with 10 g/L surfactin, resulting in an OD_600_ of 5.6, although the growth rate µ was drastically reduced during the exponential growth phase (µ = 0.186; reduction of 59%). A subsequent increase in surfactin concentration led to a substantial decrease in *B. subtilis* cell growth. Accordingly, the presence of 30, 50 and 70 g/L surfactin reduced the maximum OD_600_ values to 3.7, 2.4 and 1.5 and the growth rates µ during the corresponding exponential phase to 0.166, 0.157 and 0.149, respectively.

### 3.4. Proteomic Alterations of Unadapted B. subtilis Cells after Surfactin Treatment

Since bacterial adaptation mechanisms are induced immediately after stress induction, adaptation of the *B. subtilis* proteome to high surfactin amounts was detected with non-adapted *B. subtilis* KM0 cells, which are not capable of surfactin production. For this purpose, the mineral salt medium commonly used for *B. subtilis*-mediated surfactin production was applied. For the investigations of protein-based stress adaptation to high amounts of surfactin, as should also occur in bioreactor fermentations if possible, 75 g/L surfactin was used.

**Figure 4:**
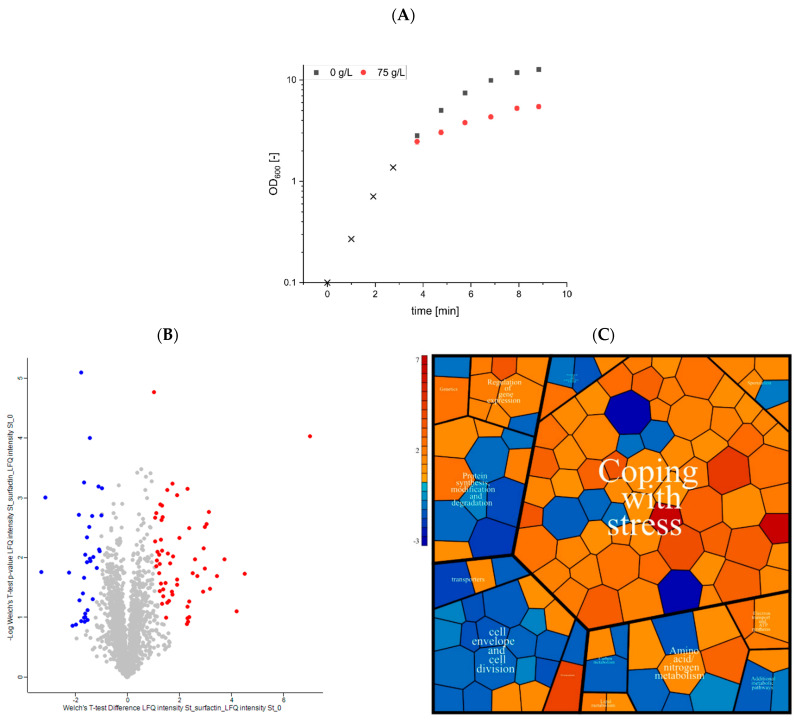
Identification and classification of proteins with altered abundance after surfactin treatment. (**A**) *B. subtilis* KM0 was cultivated in mineral salt medium (black crosses). An equal volume of the cell suspension was then mixed with either fresh mineral salt medium (black squares) or with surfactin solution (red dots). The cultivations and stress approaches were performed in biological triplicates. (**B**) Differences between protein abundances identified before and 10 min after surfactin treatment were mapped into a Volcano plot using R package *proteus* (v. 1.6.14.0). Non-significant proteins (marked in grey) are below the *p*-value range, while significantly reduced proteins were highlighted in blue and induced proteins in red. The *x*-axis shows the log_2_ fold-changes of the identified proteins between control and surfactin treatment, while the *y*-axis demonstrates the -log_10_ of the adjusted *p*-value. (**C**) All proteins with significantly changed abundances after surfactin treatment were classified according to information from the SubtiWiki database [55]. Proteins with increased presence after surfactin treatment were colored in red, while reduced proteins were highlighted in blue. The specific colouring was based on the calculated log_2_ fold-changes. The size of the polygon was determined based on the occurrence of each protein in the dataset.

While the reference process without surfactin treatment showed growth rates of approximately 0.37, a reduction about 38% was observed after addition of 75 g/L surfactin, resulting in growth rates of 0.23 (Figure 4A). To gain insight into the initial proteome adaptations, samples were taken immediately before and 10 min after surfactin treatment. Changes in the proteome were measured by NanoLC-ESI-MS/MS experiments and visualised by Volcano plots and Vonoroi treemaps (Figure 4B,C).

Overall, 38.5% of the theoretical proteome could be identified, with 3.5% (57 proteins; Figure 4B, red) significantly increased and 2.0% (33 proteins; Figure 4B, blue) reduced in abundance after addition of surfactin compared to the reference cultivation. Classification using information from the SubtiWiki database [55] in a coloured Voronoi treemap showed that a large number of 66 proteins could be assigned to the category “coping with stress”. Other classifications with relatively high proportions of proteins with altered abundances were “cell envelope and cell differentiation” (19 proteins) and “protein synthesis, modification and degradation” (11 proteins) (Figure 4C).

Specifically, genes encoding a notable proportion of the proteins significantly induced after surfactin treatment were described as targets of the alternative sigma factors σ^B^ and σ^W^. Overall, according to the SubtiWiki database [55], about 52% of all significantly induced proteins were part of the σ^B^ regulon and about 19% were regulated by σ^W^. In this context, the results also showed the induction of redox-sensitive modulators of the general stress response, Spx and MgsR, indicating the presence of intracellular oxidative stress [62,63]. Consequently, proteins associated with sub-regulatory networks, such as YsnF, YdbD, YdaD, YdaG, YhxD and YhdF, which are only detectable after redox-sensitive MgsR activation, could be measured as significantly induced (Table 2) [64].

In addition to the relevance of alternative sigma factors, the identification of LiaH as the most strongly induced protein revealed the activation of the two-component system LiaRS. Previous studies have shown that LiaRS is part of the stress response to antibiotics that are active on the cell wall (e.g., bacitracin, nisin, ramoplanin, vancomycin and daptomycin), but also to bioactive biosurfactants, such as rhamnolipids [30,65,66].

Another aspect noted after surfactin treatment was an increased formation of enzymes involved in tryptophan biosynthesis (TrpA, TrpB and TrpE). Comparable observations were previously described in heat shock experiments with *B. licheniformis* [67], suggesting that the increased tryptophan biosynthesis is part of a general bacterial stress response.

In contrast, most of the proteins classified in “cell envelope and cell division” (52% of all significantly reduced proteins) showed a reduction in their abundances after treatment with surfactin, indicating a general decrease in bacterial metabolism and thus cellular reproductive capacity. In particular, lower abundances were found for the autolysins LytC, LytE and LytF. These hydrolytically active enzymes are important for cell wall turnover and consequently for cell elongation and cell separation [68]. The reduced protein level of autolysins is consistent with the reduced growth rates calculated after addition of increasing surfactin concentrations (Figure 3 and Figure 4).

Table 2 gives an overview of the significantly changed protein abundances with differences ± 1.5.

## 4. Discussion

Since studies have described that surfactin molecules are able to integrate into lipid bilayers, leading to membrane destabilisation, pore formation and chelation [19,20,21], it has been generally assumed that surfactin possesses antimicrobial properties [15,69]. In order to analyse a negative effect of surfactin on microbial cell growth, different established laboratoy bacterial strains, namely *B. subtilis* (native surfactin producer and Gram-positive model strain), *C. glutamicum* (biotechnologically established production strain), *E. coli* (Gram-negative model strain) and *P. putida* (biotechnologically established rhamnolipid production strain), were used in surfactin shock experiments. Remarkably, the addition of surfactin produced by *B. subtilis* (KANEKA, Osaka, Japan) in concentrations of up to 100 g/L showed absolutely no or only minor effects on the respective cell growth of tested strains. With a reduction in OD_600_ values of 22 and 17%, respectively, *E. coli* and *B. subtilis* showed the greatest effects, while no considerable influence could be detected against *C. glutamicum* and *P. putida*. This raises the question of the antibacterial efficiency of surfactin. Several studies have described antimicrobial effects of surfactin in combination with other classes of lipopeptides, especially iturin and fengycin [70,71], and also physical variables, such as temperature [72]. While growth-reducing effects of *B. licheniformis*-mediated surfactin C version on *Brachyspira hyodysenteriae* and *Clostridium* perfringens have been reported [73], combinations of lipopeptides and other bioactive metabolites appear to have stronger antimicrobial effects [74]. In particular, the iturin and fengycin families appear to play an important part [75,76]. In addition to the biosynthesis of lipopeptides, *B. subtilis* is also capable to produce other bioactive and antimicrobial metabolites, such as lantibiotics and macrolides [77,78], which have additional effects on microbial fitness. Overall, surfactin seems to be more potent in its antimicrobial activity through the sum of bioactive and antimicrobially active substances.

In this study, surfactin showed growth-reducing effects in the cultivation of *B. subtilis* on a gram-per-litre scale. Overall, a reduction in maximum optical density of about 80% and in growth rate during the exponential phase of about 67% was observed between the reference cultivation and the supplementation of 70 g/L surfactin. Accordingly, it can be expected that the production of high surfactin amounts in bioreactor systems with mineral salt medium, as exemplified by Klausmann et al. [6], reduces microbial cell growth [6]. Whether specific productivity is also affected in this context should be investigated in future studies. The results of the proteome analysis already suggest that the expression of the *srfA* operon might be reduced by the increase of the Spx regulator, which acts as a repressor for the *srfA* operon [79]. Contrary to this assumption, for example, is the σ^B^-mediated stimulation of the *srfA* operon expression during the general stress response [23]. However, no SrfA subunit was identified with a significantly altered protein abunadance after surfactin stress. Nevertheless, further analyses on possible post-translational feedback mechanisms of surfactin on lipopeptide biosynthesis needs to be addressed in the next studies.

However, relatively high scale values in grams per litre were required to detect effects. In contrast, the treatment of rhamnolipids and sophorolipids as further exemplary microbially produced bioactive metabolites showed significantly lower minimal inhibitory concentrations. In this context, bacterial growth behavior was affected in different ways. While treatment with rhamnolipids induced cell lysis in *B. subtilis*, growth-reducing and bacteriostatic effects were observed in *P. putida* and *E. coli*, respectively. Based on the observation that rhamnolipids appear to have a negative feedback loop to *P. putida*, a natural limitation of heterologous rhamnolipid production with *P. putida* as host seems conceivable. In the case of sophorolipids, similar observations were made for *B. subtilis* (cell lysis) and *C. glutamicum* (inhibition of cell growth), while Gram-negative *E. coli* and *P. putida* were not affected in their growth using concentrations of up to 1 g/L. Consequently, both the mode of action and the antimicrobial efficiency of the respective bioactive metabolite seem to depend on the genus of the target organism.

To get more knowledge about the response of *B. subtilis* after treatment with high amounts of surfactin, surfactin shock experiments were performed in mineral salt medium. Subsequent analysis of the proteome alteration confirmed the hypothesis of a reduction in growth as evidenced by the reduction of autolysins, which are involved in cell wall turnover during cell elongation and separation. Furthermore, proteomic data showed that *B. subtilis* activates both a specific stress response to protect the cell surface (σ^W^) [80,81,82] and a non-specific and multiple stress resistance (σ^B^) [83]. In this context, redox-sensitive modulators of the σ^B^ regulon, Spx and MgsR were induced, suggesting the stimulation of both cell wall stress and oxidative stress, which could be caused by surfactin-mediated damage to the cell membrane [62,63]. Additionally, the strong induction of LiaH indicates activation of the LiaRS two-component system, suggesting that surfactin-mediated cell surface penetration triggers a stress response comparable to that of other antibiotics that are active on the cell wall, such as bacitracin, nisin, ramoplanin, vancomycin and daptomycin [65,66].

Another cyclic lipopeptide described with antimicrobial properties, friulimicin B, produced by *Actinoplanes friuliensis*, shows inhibitory properties against peptidoglycan synthesis at sublethal amounts of 1 μg/mL [84,85]. Results by Wecke et al. showed that friulimicin B induces σ^M^- and σ^V^-mediated gene expression in *B. subtilis* [28]. In contrast to surfactin, activation of σ^W^ and LiaRS two-component system was not observed, suggesting that the cyclic lipopeptides produced by different bacteria differ in their interaction with the cell surface of *B. subtilis* and therefore stimulate different stress response mechanisms. This assumption was confirmed by comparable transcriptome analyses with friulimicin B and the structurally similar cyclic lipodepsipeptide daptomycin. While a clear LiaRS induction could be measured after daptomycin treatment, no activation was detectable for friulimicin B [28].

## 5. Conclusions

Partial growth-influencing properties of surfactin on different bacterial production organisms were, if at all, only detectable after additions in the order of several grams per litre, which was significantly less effective compared to the other bioactive metabolites rhamnolipids and sophorolipids. These findings are in strong contrast to the general assumption that surfactin has relatively pronounced antimicrobial properties. Accordingly, surfactin appears to develop antimicrobial properties only in synergy with other antimicrobial compounds. However, since the use of a chemically defined mineral salt medium dramatically increased the surfactin-mediated effect on *B. subtilis* growth rates, surfactin could also have a significant impact on bioreactor processes. The open question of whether general microbial productivity is affected by the slowed cell growth needs to be addressed in further studies. Furthermore, the open question of the impact of a synergistic effect of surfactin with other simultaneously produced antimicrobial compounds on native surfactin production should be answered in the future. Nevertheless, the results show that *B. subtilis* is able to tolerate significantly higher surfactin titres than those previously described in bioproduction processes. Accordingly, an increase in bacterial surfactin production to more than 100 g/L does not seem to be a limit from the perspective of a negative feedback inhibition of surfactin on *B. subtilis* growth. A doubling or even a quadrupling of the currently described highest titres therefore seems possible and would facilitate an economic establishment of surfactin as a surfactant alternative on the market.

The investigation of an initial surfactin stress response of *B. subtilis* revealed the induction of different regulatory networks associated with the alternative sigma factors σ^W^ and σ^B^, but also the LiaRS two-component system. Consequently, the activation of both cell wall-specific and general stress responses by surfactin treatment is involved. These findings will help to develop more robust *B. subtilis* surfactin production strains, potentially leading to increased product-per-biomass yields.

## Figures and Tables

**Table 1:** Overview of the strains used in this study.

Strain	Genotype	Reference	Surfactin Conc. [g/L] Used
*Bacillus subtilis* KM0	168; *trp^+^*	[43]	10 ^#^, 30 ^#^, 50 *^,#^, 70 ^#^, 100 *
*Corynebacterium glutamicum* ATCC13032	wild-type	[44]	50 *, 100 *
*Escherichia coli* BL21 (DE3)	F– *omp*T *hsdS*_B_ (rB–, mB–) *gal dcm* (DE3)	Thermo Scientific^TM^	50 *, 100 *
*Pseudomonas putida* KT2440	r^−^ m^+^	[45]	50 *, 100 *

* Surfactin was used in LB medium. ^#^ Surfactin was used in mineral salt medium.

**Table 2:** Proteins significantly affected in their abundance after surfactin treatment. Proteins were only listed with differences ≥1.5 or ≤−1.5.

Protein Name	Log2 Fold Change	Regulators	Functions, Homologies
Increased after surfactin treatment (≥1.5)			
LiaH	7.02	LiaRS	resistance against oxidative stress and cell wall antibiotics
YdaD	4.51	σ^B^	general stress protein (similar to alcohol dehydrogenase)
ZagA	4.20	Zur	zinc metallochaperone
YsnF	3.75	σ^B^	general stress protein
MgsR	3.45	σ^B^	modulator of general stress response
CsbD	3.18	σ^B^	general stress protein
YbyB	3.15	σ^B^	general stress protein
YhdF	3.04	σ^B^	similar to glucose 1-dehydrogenase
GspA	2.99	σ^B^	general stress protein (similar to glycosyl transferase)
YbfO	2.98	σ^W^, AbrB	similar to erythromycin esterase
YjgD	2.94	σ^B^	general stress protein
TrpE	2.92	MtrB	anthranilate synthase (tryptophan biosynthesis)
YjzH	2.69		unknown
YjgC	2.60	σ^B^	general stress protein (formate dehydrogenase)
YdbD	2.52	σ^B^	general stress protein (similar to manganese-containing catalase)
RpmF	2.39		ribosomal protein
YflT	2.38	σ^B^	general stress protein
YxaB	2.38	σ^B^, AbrB	general stress protein (similar to pyruvyl transferase)
Rtp	2.35		replication terminator protein
YurQ	2.33		unknown
YcdF	2.32	σ^B^	general stress protein (similar to glucose 1-dehydrogenase)
YflH	2.31	σ^B^, NagR	general stress protein
YhxD	2.30	σ^B^	general stress protein (similar to alcohol dehydrogenase)
YdaG	2.00	σ^B^	general stress protein (putative pyridoxamine 5′-phosphate oxidase)
YbfP	1.93		similar to transcription factor (AraC family)
Spx	1.91	σ^B^, σ^W^, σ^M^, σ^X^, PerR	transcriptional regulator
YrhJ	1.91	σ^W^, σ^M^, σ^X^, FatR	cytochrome P450/NADPH-cytochrome P450 reductase
RpmE2	1.76	σ^B^, Zur	general stress protein, accessory ribosomal protein under zinc limitation
YuaI	1.73	σ^W^	unknown
OhrB	1.73	σ^B^	general stress protein
NhaX	1.73	σ^B^	general stress protein (putative regulator of NhaC)
PadR	1.64		regulator of the phenolic acid stress response
YoxC	1.61	σ^B^	general stress protein
McsA	1.57	σ^B^, σ^M^, σ^F^, CtsR, Spx	modulator of CtsR regulator
YdjP	1.55	σ^W^ σ^E^	similar to chloroperoxydase
YsmB	1.54		similar to transcriptional regulator (MarR family)
YhcW	1.50		putative glycerol-3-phosphatase
decreased after surfactin treatment (≤1.5)			
YxxD	−3.29		antitoxin
CspC	−3.15		RNA chaperone
SecG	−2.24	SigB	preprotein translocase subunit
YdgH	−2.11	LexA	similar to drug exporter
BdbA	−1.96	Rok, Abh, DnaA, YvrHb, AbrB	thiol-disulfide oxidoreductase
LytE	−1.86	σ^H^, σ^I^, WalR, Spo0A	cell wall hydrolase (major autolysin, cell elongation, separation)
YfkK	−1.83	σ^H^, σ^I^, Spo0A, WalR	cell wall hydrolase
CysE	−1.77		serine O-acetyltransferase
LytC	−1.76	σ^D^, SinR, YvrHb, SlrR	N-acetylmuramoyl-L-alanine amidase
SdpI	−1.70	AbrB, SdpR	immunity protein
YkfB	−1.67	CodY	L-Ala-D/L-Glu epimerase
PanD	−1.65		aspartate 1-decarboxylase
YckB	−1.65		similar to amino acid ABC transporter
BceA	−1.62	BceR	ABC transporter for target protection of cell wall synthesis
CwlS	−1.61	σ^D^, σ^H^, CcpA, Abh, AbrB	D,L-endopeptidase; peptidoglycan hydrolase
MntH	−1.60	MntR	manganese transporter
RbsC	−1.60	CcpA, AbrB	ribose ABC transporter
LytF	−1.55	σ^D^, SlrR, SinR	gamma-D-glutamate-meso-diaminopimelate muropeptidase
YoeB	−1.55	WalR	inhibitor of cell separation and autolysins
YurK	−1.53		transcriptional regulator (GntR family)
TcyB	−1.52		cystine and diaminopimelate ABC transporter

## Data Availability

The data presented in this study are available on request from the corresponding author. The mass spectrometry proteomics data presented in this study are openly available in the PRIDE partner repository with the dataset identifier PXD029668.

## Supplementary Materials

The following supporting information can be downloaded at: https://www.mdpi.com/article/10.3390/microorganisms10040779/s1.
